# The challenges that individuals with MDRTB and TB experience when returning to work after completing TB treatment in the Western Cape, South Africa

**DOI:** 10.1177/10519815251330123

**Published:** 2025-04-17

**Authors:** Mogammad Shaheed Soeker, Ayesha Jainodien, Mario Smith

**Affiliations:** 1 Occupational Therapy Department, University of the Western Cape, Cape Town, South Africa; 2 Psychology Department, University of the Western Cape, Cape Town, South Africa

**Keywords:** adaptations, client-centred approach, experience, occupational therapy, perception, qualitative research, return to work, self-efficacy, tuberculosis, vocational rehabilitation and young adults

## Abstract

**Background:**

Tuberculosis (TB) and multi-drug resistant (MDR) TB have been identified as one of the largest health problems in the world, and notably recognised as a major concern in South Africa. Socio-demographic factors such as poverty and unemployment has been identified as major contributing factors to the epidemic.

**Objective:**

The aim of the study was to explore the barriers and enablers of return to work (RTW) for individuals living with MDRTB and Pulmonary Tuberculosis (PTB) in the Western Cape, South Africa. The qualitative exploration was part of a larger study that resulted in a return-to-work programme for individuals with MDRTB and TB.

**Method:**

Qualitative methodology was employed for this study. Semi-structured interviews were used with 5 key informants and 4 participants diagnosed with PTB and MDR-TB. The data was analysed by means of thematic analysis.

**Results:**

The participants described the barriers and facilitators of their RTW process and the factors that were of critical importance in the resumption of the worker role. Two themes represent the barriers, and two themes represent the enablers of RTW. The four major themes were 1) A sense of disbelief in one's own potential, 2) The contextual environment influences an individual's worker role, 3) The future of Occupational Therapy (OT) services in TB rehabilitation, and 4) Engagement in activities alleviates feelings of despondency.

**Conclusion:**

The study highlighted that contextual barriers such as poor socio-economic factors negatively influenced individuals diagnosed with PTB/MDRTB from initiating and completing rehabilitation programmes. The lack of resources in public facilities were also noted as one of the main barriers described by the rehabilitation care specialists. The facilitators that supported the completion of rehabilitation programmes enhancing the life skills of and work related skills as part of the PTB/MDRTB intervention provided to clients or patients. Early engagement in work skills improvement programmes may enhance the worker roles of individuals with PTB/MDRTB.

## Introduction

The World Health Organisation^1^ describes tuberculosis as an infectious bacterial disease that is caused by mycobacterium tuberculosis, which most commonly affects the lungs of individuals. Rossouw,^2^ has stated that there are 2 billion people worldwide who are infected with TB, and this alarming figure is increasing dramatically, as there are 8 to 9 million new cases each year. Eighty percent of the new cases of TB are found in the twenty-two highest burden countries.^2^ According to the WHO,^3^ 87% of new TB cases occur in the 30 high TB burden countries, with South Africa being one of the eight, high TB burden countries.^3^ TB is therefore a well-known medical condition in South Africa due to its growing incidences. With South Africa being 9^th^ on the list of 22 “high burden countries” that are most affected by TB, we should be concerned about the future population of this country as this statistic is alarming.

The term incidence refers to the measure of probability of the occurrence of new cases of disease over a specific period of time, which is commonly measured as a rate or proportion.^4^ As stated by the WHO, about 8.6 million cases were recorded in 2012, with the highest incidence occurring in Asia and Africa.^5^ Baitsiwe^6^ states that South Africa has the 5th highest TB incidence in the world with 0.46 million cases reported in 2007.^7^ The incidence of TB in South Africa, estimated by the World Health Organisation to be 468 per 100,000 of the population in 2022, this remains very high.^8^ South Africa runs the world's largest HIV treatment programme with 5.4 million individuals out of an estimated population of 60.4 million currently receiving antiretroviral therapy, no less than 54% of the estimated incident TB cases and 57% of the 54,000 deaths attributable to TB in 2022 occurred in HIV-infected persons.^9^

### NB: For noting the current paper was originally part of one of the co-author's thesis

It can be assumed that individuals living with TB form a large percentage of the working population. As a result, it is important that adults living with TB continue to engage in work skills that they are capable of performing. According to the South African National Tuberculosis Control Program Practical Guidelines,^10^ one of the most common symptoms of pulmonary TB is the loss of motivation in individuals. Motivation is the driving force behind one's source of willingness to engage in everyday activities. Therefore, if adults living with TB suffer from a loss of motivation their general confidence in their work skill abilities become affected.

Occupation of work for individuals suffering from TB is affected as they have poor work skills due to their illness.^11^ The above could cause that individuals diagnosed with TB to experience stigma and low self-esteem. Low self-esteem may cause these individuals to struggle to maintain work when they get re-infected. This is due to the fact that TB infected individuals perceive themselves to be at risk of a number of social and economic consequences due to stigma.^11^ Courtwright and Turner^12^ discussed that due to the fact that the most common result of the TB stigma is isolation from other members of society, therefore the TB disease can have a great impact on economic opportunities for TB survivors. For example, the stigmatisation of TB in Ghana has led to the banning of TB survivors from selling goods in public markets and attending community events. This type of isolation and stigmatisation from society has an impact on the socioeconomic and financial status of individuals. Men in general, are more concerned about the impact of the TB stigma on their financial prospects, which include job loss and reduced income.^12^

## Methods

The qualitative research methodology was used in order to describe the experiences of survivors of cardiac disease about returning to work after participating in a cardiac rehabilitation programme. The sampling technique utilised was purposive sampling in order to select the participant. Purposive sampling enables the researcher to select research participants based on their characteristics as it relates to the study.^13^ This type of sampling was suited for the study as all the participants who attended the TB programme at a TB specialised care hospital were eligible to be selected as part of the sample. Nine participants who were willing and available were selected to participate in the qualitative strand of the study.

**Table 1.** Description of the demographics of participants for semi-structured interviews.

Key informant (K)	Age	Gender	Qualification	Years of experience working as an Occupational Therapist
K 1	25	Female	Bachelor degree Occupational Therapy	3
K 2	30	Female	Bachelor degree Occupational Therapy	8
K 3	32	Female	Bachelor degree Occupational Therapy	10
K 4	28	Female	Bachelor degree Occupational Therapy	6
K 5	30	Male	Bachelor degree Physiotherapy	6

Participant	Age	Gender	Primary diagnosis description	Education	Pre-MI occupation	Classification of occupation	RTW after rehabilitation
1	35	Female	PTB	Secondary	Teller	Light	Yes
2	53	Female	PTB	Secondary	Support group facilitator	Medium	Yes
3	54	Female	MDRTB	Tertiary	Nurse	Medium	Yes
4	40	Male	MDRTB	Secondary	Factory worker	Medium	Yes

**Table 2.** Themes and categories.

**Theme one**	**Categories**
A sense of disbelief in one's own potential.	The negative influence of TB on an individual's self- belief The negative influence of substance abuse on treatment adherence and an individual's self- belief
**Theme two**	**Categories**
The contextual environment influences an individual's worker role	Poor socio economic conditions influence the worker role Work opportunities in the community influences the worker role
**Theme three**	**Categories**
A sense of disbelief in one's own potential.	Developing OT services that aid in work place reintegration Rehabilitation services in primary health care
**Theme four**	**Categories**
A sense of disbelief in one's own potential.	Participation in activities regulates negative emotions related to returning to the worker role Engagement with others, facilitates socialisation

## Participants

There are two population groups that were sampled for this study. The first population represented the key informants working in the private and/or public sector as rehabilitation specialists. The second population group for this study were individuals living with PTB and MDR-TB who have returned to work. A **sample of five rehabilitation specialists** were recruited using the following inclusion criteria: For inclusion in this study, eligible participants had to be health rehabilitation experts such as Occupational therapists or Physiotherapists, who have worked with PTB or MDRTB clients for at least 6 months and have were involved or experience in getting PTB and MDRTB clients back to work. The exclusion criteria for the rehabilitation specialist was that rehabilitation specialists who were only overseeing rehabilitation programme but not treating individuals with PTB and MDRTB were excluded from the study.

The second population group for this study consisted of four **individuals living with PTB and MDR-TB** who have returned to work. Inclusion criteria: for inclusion in this part of the study it was essential that the participants had been previously diagnosed with PTB and MDR-TB.

The exclusion criteria were that individuals with PTB and MDR TB who had active symptoms or were infectious were excluded from participating in the study. A sample size of between 5 to 25 is regarded as appropriate for qualitative studies as the purpose of qualitative studies is to obtain and in depth understanding of phenomenon.^13^ The sample of 9 individuals was therefore regarded as appropriate for the study. The purpose of the study was to under the experiences of individuals with PTB and MDRTB about returning to work after they have completed rehabilitation (Table 1).

## Procedure

The participants were identified through the statistical records held at public hospitals, where they were contacted via telephone. Through a telephone call, the participants were given brief information about the study, and the researcher ensured that the participant met the criteria for inclusion, and then arranged an appointment for discussing possible participation and interest. Thereafter, an introductory meeting with each participant took place where the aim of the study was explained both verbally and in writing. The participants’ informed consent was sought and obtained, after which interview dates were arranged. All of the introductory meetings and interviews took place in the Occupational Therapy department at the TB/HIV care association and in public hospitals. During data collection, semi-structured interviews were conducted, where the researcher had the opportunity to prompt for responses that aided in obtaining rich information. The duration of the interviews was between 45–60 min.

## Data collection

Two individual semi-structured interviews were completed with the 05 key informants and 4 participants about returning to work after they have completed their rehabilitation. The data was collected at the hospital site where the rehabilitation specialists were employed and where the individuals with PTB and MDRTB received treatment.

## Analysis and trustworthiness

In this study, Tesch's eight steps of data analysis guided the analyzing of the data. Tesch believes that data analysis is an eclectic process.^14^ The process commenced with reading all the transcripts, noting down ideas that were emerging. This was proceeded by the selection of one interviewee's transcript at a time to extract meaning from the information. This resulted in similar topics being grouped together. Abbreviating the topics as codes, with the codes being noted next to the appropriate segment of text, the organised data led to new categories and codes emerging. Descriptive wording for the topics was used to develop categories. The abbreviation of each category and code was arranged alphabetically. Preliminary analysis was performed with the information from each category. The process concluded with the data being recorded. The researchers followed an inductive research process in order to develop, the three themes that emanated from this study. The researchers used the strategies as advocated by Krefting^15^ in order to facilitate trustworthiness, namely, four basic criteria namely truth value, applicability, consistency and neutrality of data. The researcher will make use of member checking, the provision of detailed descriptions of the research process and triangulation.

## Ethics

The WHO^16^ ethical guidelines promoted the ethical conduct of research, through the enhancement and protection of the rights of the research participants. Ethics approval was sought from the University of Western Cape's Research Ethics Committee before the commencement of the study. An information sheet was prepared, which included details regarding the study being conducted, information regarding confidentiality, the risks and benefits involved. The researcher ensured anonymity through the use of pseudonyms and kept transcribed data locked in a password-protected computer.

## Results

The participants described the barriers and facilitators of their RTW process and the factors that were of critical importance in the resumption of the worker role or the result of unemployment. Two themes represent the barriers, and two themes represent the enablers of RTW. The four major themes were 1) *A sense of disbelief in one's own potential,* 2) *The contextual environment influences an individual's worker role*, 3) *The future of OT services in TB rehabilitation*, and 4*) Engagement in activities alleviates feelings of despondency *(Table 2)*.*

### Theme One: A sense of disbelief in one's own potential

The above theme describes the participants’ perceptions of their abilities following their diagnosis of PTB and MDR-TB.

The participants interpreted the lack of physical and functional abilities one has when diagnosed with TB or MDR-TB as a burden that causes you to be demotivated. The lack of motivation and emotional instability leads to a disbelief in their potential. One participant captured the description by saying:

“*you will get the very motivated patient who will think, you know I’m here now but I’m going to make the most of my time, but then you will get those who don’t want to do anything because they feel that they’re just there for their medication so they don’t really want to participate in anything else and then you get some that are just too sick to participate in like group therapy.” (P1: Rehab specialist)*

“*it's difficult because you have to really motivate them” (P1: Rehab specialist)*

*“I didn’t want to go to OT but when time goes on then I did want to go to OT.” (P2: TB survivor)*

The above quotes illustrate that it is common for TB and MDR patients to withdraw from participation in task and activities, either due to lack of motivation or physically, due to poor endurance. This feeling of helplessness and lack of motivation eventually results in a loss of confidence in their ability. The following categories are used to discuss the foregoing theme: the negative influence of TB on an individual's self-belief, and the negative influence of substance abuse on treatment adherence and an individual's self- belief.

#### The negative influence of TB on an individual's self- belief

The above category describes the participants’ perception of how TB or MDR-TB negatively impacts the TB survivor's level of self-belief. The loss of belief in oneself often results in feelings of despondency, and the manner in which a TB or MDR- TB diagnosis can cause a change in oneself, eventually affecting engagement in daily activities and vocational skills. One participant stated

“*Some are actually dragged into the hospital to try and get them to finish their medication those are usually the ones that are not motivated” (P3: Rehab specialist)*

#### The negative influence of substance abuse on treatment adherence and an individual's self- belief

This category represents the role substance abuse plays in the treatment adherence of individuals with PTB and MDR-TB. The category also describes the participants’ experience and perception of the negative influence that substance abuse has on an individual's self-belief. One of the participants described her experience by stating:

“*a lot of the things now that are being covered is aimed at treatment adherence and not using substances and finishing your treatment.” (P1: Rehab specialist)*

Another participant indicated that PTB and MDR-TB survivors have difficulty with adhering to the treatment protocol once they are discharged from the hospital, and forget what they promised themselves when they were admitted to hospital. She said:

“*When they get outside or they just relapse when they get outside and then they just continue their previous lifestyle.” (P2: Rehab specialist)*

### Theme Two: The contextual environment influences an individual's worker role

The above theme represents the barriers identified by participants which influences their worker Role. Participants described the common worker role of patients who are admitted to hospital, the nature of the survivor's job characteristic, and stigma from society. Participants indicated how important the context of where the PTB or MDR-TB patient comes from, and how the context can influence your motivation to return to work. One participant said:

“*If you find a patient that comes from a secured community where it's frowned upon to not work with that patient particularly he will probably want to go to work” (P3: Rehab specialist)*

#### Poor socio economic conditions influence the worker role

Under this category, participants elaborated on the external factors in the community where they influence patients diagnosed with PTB and MDT-TB. It appears to be a common phenomenon for people to motivate for disability grants instead of pursuing the idea of returning to work. Most participants were of the opinion that because of their diagnosis they will never be able to return to work. One participant captured this description by stating:

“*They’re all interested in a disability grant and that is their focus, some of them think that they’re too weak, they’ll never be able to work again” (P2: Rehab specialist)*

This category is explained further with subcategories relating to ineffective community reintegration and the abuse of social support grants.

#### Work opportunities in the community influences the worker role

This category explains the participants’ description of the worker role in the community. Participants in this study expressed that it was challenging to encourage PTB and MDR-TB survivors to be motivated towards work related activities while in the rehabilitative phase of treatment, as some of them might not have worked prior to being admitted to hospital. One participant described this by stating:

“*then with the substance abuse then maybe they were not even ever working before so a lot of work programs are aimed at ‘Okay so this is what is meaningful to you and this is what you use to do but how can we adjust what it is that you are doing so that when you go out you can work in something that's more suited to your abilities now’ but they don’t come from a culture of working so now you’re trying to create a whole new culture and that I think is a big challenge as well…”(P1: Rehab specialist)*

### Theme three: The future of OT services in TB rehabilitation

This theme represents the participants’ perception of change, and how a change in service delivery can facilitate improved OT services that specialise in the treatment of TB. The participants indicated that work related intervention programmes would enable the PTB and MDR-TB survivors to re-engage in their worker role more effectively. Engagement in work related programmes will also improve their ability to return to work and grant those who are unskilled to learn new skills. One participant said:

“*OT doesn't have to be me sitting here and making a little crafty thing, but that you can make a crafty thing in the community, but it can be how to use a computer because that is something that every job now, you need to know how to use a computer” (P1: Rehab specialist)*

#### Developing OT services that aid in work place reintegration

This category focuses on the return to work of PTB and MDR-TB patients, and how OT services can assist in this process. One participant stated:

“*When we did focus groups, the requests was to have uhm ‘how to, how can we find a job” (P2: Rehab specialist)*

Another participant described the programmes and activities that are currently being facilitated in government run TB institutions.

“*At the moment we have education, education is: HIV, TB, eating healthy, get your life back, like the goal setting uhm life skills, time management uhm stress management, conflict all of those life skills stuff. Uhm and then there's leather work and then there's arts and crafts and then there's substance use groups” (P2: Rehab specialist)*

- **A need for vocational rehabilitation programmes in existing TB programmes.**

This category describes how vocational rehabilitation programmes can improve rehabilitation service in order to facilitate the return to work of PTB and MDR-TB survivors. The participants were of the opinion that providing vocational rehabilitation in TB programmes would enhance the return to work rates of TB survivors. One participant stated:

“*the gap has always been with work and simulating appropriate tasks for the patient to prepare them to go out there and actually engage uhm you know in the community” (P1: Rehab specialist)*

Another participant stated:

“*Currently we do not have uhm a set program for the clients to return to work” (P3: Rehab specialist)*

#### Rehabilitation services in primary health care

This category indicates that the engagement of participants in vocational rehabilitation programmes will facilitate the resumption of the TB survivor's worker role, as well as encourage holistic occupational therapy practice in a TB rehabilitation setting. She said:

“*It will definitely add benefit, it brings the occupation (work skills retraining) back, whereas now sometimes the way OT is, you get sidetracked based on the culture of the institution you are at, but this brings it all back to occupation so I think it can definitely be used and be woven into what is already been happening” (P1: Rehab specialist)*

- **Enhance vocational rehabilitation to allow for early intervention**

This subcategory conveys that through improving vocational rehabilitation, TB survivors will receive early intervention, which will enhance the understanding of the TB disease, and that returning to their previous worker role is possible. One of the participants indicated:

“*Equip them just to make sure they understand that it's not the end of road if you have the disease. It's just, you must make sure that you are going to finish your treatment.” (TB2: TB survivor)*

### Theme Four: Engagement in activities alleviates feelings of despondency

This theme describes the participants’ experience and perception of engagement in activities, and how it alleviates feelings of despondency during rehabilitation. The participants in this study revealed that engaging in activities led to an improvement in the medical and emotional state of the TB survivors. They further indicated that this improvement in health brought about positivity and was an encouragement to resume their previous worker role. One participant indicated this by stating:

“*I used to do that and then each and every day even on Sundays I have to go to OT and then doing the program, for me it was fine and good because it made me feel comfortable and then believed in myself.” (TB2: TB survivor)*

#### Participation in activities regulates negative emotions related to returning to the worker role

This category describes how the participants’ engagement in activities during the rehabilitation phase of treatment helped facilitate their recovery through decreasing their negative emotions, and ultimately aided in the resumption of their worker role. One participant said:

“*I went to OT out of my own, I just told myself I need to get up and help myself, and if I can, help other people in the ward” (TB1: TB survivor)*

Another participant said:

*Yes I did achieve many goals because I did set the goals, I remember the time I was admitted there I said: ‘ I’m going to achieve the goals’ and then I said: ‘No the first week I must finish this, I must do that’ and now I finish this (work task), and then the second week and then by the end of the month I finished this (work task) and then I start the other one, I always keep the goals then I finish the six months and everything was fine for me because I always when I wake up I just said: ‘No I’m going to finish this day and then my goal I’m going to finish it’. (TB2: TB survivor)*

#### Engagement with others, facilitates socialisation

This category focuses on the influence other clients/patients can provide during the rehabilitation phase through creating a supportive environment. It is evident through this statement that it only takes one motivated participant to encourage and support others. One participant indicated this by stating:

“*Maybe they scared because they don’t want to come here, they stay in their rooms but I encourage them, I go and encourage them to come here.” (TB3: TB survivor)*

## Discussion

In the following section the enablers and barriers that affected the individual living with TB/MDRTB in returning to work will be discussed.

### Barriers to return to work

Themes One and Two addressed thematic content related to barriers to returning to work.

#### A sense of disbelief in one's own potential

As a result of the findings in this study, there were many barriers identified by participants, one of which was, “A sense of disbelief in one's own potential”. This barrier was described in theme one of the study, where participants expressed that they experienced having poor motivation and belief in themselves after having suffered from PTB and MDR-TB.

According to the World Health Organization,^17^ barriers are more than just a physical obstacle. Rather, it is described as components in a person's environment that through its presence or absence, may hinder one's ability to function optimally within the environment. These components are described as obstacles that cause inaccessibility to one's physical environment i.e., shortage of assistive devices, society's attitude or approach towards disability, and lastly, legislation that prevents the involvement of all people. The participants expressed that being diagnosed with PTB and MDR-TB hinders one's physical and functional abilities to the extent that one starts feeling demotivated, which leads to feelings of disbelief in their potential. Even though the participants show the potential to learn new skills or perhaps return to their previous worker roles, their lack of belief in themselves limits their capacity to do so.

##### The negative influence of TB on an individual's self- belief

A participant mentioned that at times patients are forced by their families to remain in hospital in order for their course of medication to be completed. This is largely due to patients being unmotivated and showing signs of despondency. Another participant pointed out that patients often go through a lot of psychological changes when they are diagnosed with PTB and MDR-TB, such as depression and anxiety. Their feelings of despondency are often brought on by the fact that the patient could face losing their job, which would result in a loss of income and job security.^18^ These feelings of despondency also affect the PTB and MDRTB individual to not complete their medication regime. It is important to note that treatment adherence in patients with TB is a major factor in the success of their treatment. Poor treatment adherence leads to a greater chance for an individual to experience a relapse.^19^

The findings reflected in this theme are consistent with research on adherence to other syndromes where psychological processes come into play. For example, Kakili^20^ reported that mental conditions such as depressive disorders, anxiety and stress have a negative effect on treatment adherence of patients. In a study conducted on depression in South Africa, researchers found that patients with depression default from treatment more than non-depressed patients.^21^ Furthermore, it is indicated that patients who are receiving chronic medication for TB, diabetes, HIV/AIDS and hypertension tend to suffer from stress and depression as they have to adjust to coping with taking medication on a daily basis.^21–23^ Thus, the recognition of psychological sequelae in the rehabilitation of PTB patients is very important.

##### The negative influence of substance abuse on treatment adherence and an individual's self- belief

According to Kakili,^20^ TB treatment adherence in patients is an important facilitator in achieving treatment success, as poor adherence leads to a greater chance of defaulting off treatment. This will result in a greater opportunity for drug resistance, such as MDR-TB and XDR-TB. Alcoholism is another major barrier in TB adherence as excessive alcohol consumption is known as one of the major contributing factors for patients defaulting from their treatment.^19^ This is due to the fact that patients either forgot or they do not prioritise taking their medication due to intoxication. In addition, when patients default off their medication, there is a negative effect on the healthcare system as the patient will need to restart their medication regime. Kakili^20^ further states that in many developing countries such India, Mexico and South Africa, alcohol use is the major barrier to TB treatment adherence. Excessive alcohol consumption has been noted as a major factor contributing to defaulting on TB treatment. Studies found that patients tend to forget or ignore taking their medication after excessive consumption of alcohol.^24,25^

One participant stated that clinicians working in TB settings are starting to focus more on treatment adherence, as participants often relapse and default off their medication when they are discharged from the hospital. Previous lifestyles and habits are resumed, as they forget the promises they made themselves when they were hospitalised. When individuals engage in substance abuse, it naturally alters their state of mind, which can lead to poor treatment compliance. Substance abuse also affects the quality of life of individuals, as the use of substances becomes the priority in the individual's life, which results in them neglecting their health.^26^ Poor adherence to medication is one of the factors that can increase the likelihood of patients relapsing.^27^

#### The contextual environment influences an individual's worker role

**Theme Two** - “The contextual environment influences an individual's worker role” was identified as a barrier by participants in this study. This barrier was pointed out by various participants who expressed that the context in which the participants come from i.e., either their home or work environment, plays a part in determining the worker role of the participants. The participants’ context can either positively or negatively influence the participants’ desire to return to their previous worker role, based on the stigma and influence within society.^20^

##### Poor socio economic conditions influence the worker role

The participants in this study indicated that socio economic factors influence the worker roles of participants, as people who come from poor socio-economic areas tend to have a different challenges that affect their worker role when compared with individuals who come from a higher socio-economic area. However, it could be argued that it is a societal norm to seek work to earn an income. It is known that unemployment is a critical problem in many developing counties such as South Africa.^20^ Participants expressed that upon their return from areas of a lower socio-economic status, they are exposed to many social ills and stigma, and lower income jobs are less likely to employ you if you previously suffered from a disease. This has further been expressed in a study that explored the experiences of patients with adverse-drug effects of MDR-TB in primary health care facilities in the Western Cape. There are various socio-economic factors which typically impact MDR-TB patients. They are challenged in being stigmatised for having MDR-TB and often face socioeconomic barriers such as inaccessibility of accessing treatment due to transport costs, and acquiring a social grant. Participants expressed their frustration of lost income due to their incapacity of returning to work, due to the long duration of treatment. The loss of income negatively impacts the family especially when the patient is the breadwinner.^28^

##### Work opportunities in the community influences the worker role

A participant in this study conveyed that many of the patients that are admitted for PTB and MDR-TB treatment were either unemployed or they were low income workers prior to being admitted to hospital. Discrimination is a key work- related factor that surrounds PTB and MDR-TB patients, as they feel that disclosing their TB status to their employers and colleagues will allow for discrimination in the workplace. In addition, there is a fear of job loss.^20^ This ‘fear of job loss’ which most TB and MDR-TB survivors live with, is further discussed by Tinzi,^28^ where it is indicated that when a diagnosed MDR-TB patient starts their treatment, they embark on a two-year treatment regime which involves several hospital visits and close monitoring for at least the first six months of treatment. Unfortunately, not all work environments can accommodate or tolerate staff from missing out on their work responsibilities.

### Objective two: Facilitators of return to work

Themes Three and Four addressed facilitators to return to work.

#### Theme 3: future of OT services in TB rehabilitation

Theme three “The future of OT services in TB rehabilitation” was interpreted as a facilitator by the participants of this study. The WHO^29^ describes a facilitator as being a component that stimulates, promotes or enhances the surrounding environment with the aim of improving it. In this study, the participants’ perception of change described the specific facilitators that would elicit change, in guiding and showing that there is room for improvement when it comes to TB rehabilitation services, such as OT.

##### Developing OT services that aid in work place reintegration

Participants of this study revealed that the OT programme for PTB and MDR-TB rehabilitation patients does not only need to be arts and craft type activities, but activities that focus more on work rehabilitation. Work-related intervention programmes are more appropriate and suitable for PTB and MDR-TB survivors who want to return to work after rehabilitation, as this would allow them the opportunity to re-engage in their worker role more effectively. Research showed that rehabilitation interventions such as occupational therapy, physical therapy and vocational rehabilitation among others, have been reported in the literature to improve recovery and return to work.^30,31^ Rehabilitation specialists described that in government run rehabilitation settings, they have a shortage of staff capacity in order to facilitate more programmes than what they currently have in place. This therefore limits them from practicing a holistic approach to the type of rehabilitation programmes they are able to facilitate. Some participants felt strongly that if PTB and MDR-TB participants engage in a vocational rehabilitation programme, it would facilitate the resumption of their worker role, as well as encourage the practice of holistic occupational therapy. This will also provide the opportunity for early intervention, enhancing the individual's understanding of the TB disease, to the extent that they will understand that returning to their previous worker role is achievable.

##### Rehabilitation services in primary health care

The primary health care system is one that is widely known and recognised. The WHO^32^ has stated that this system focuses on providing basic health care that is both practical, scientifically based, and uses appropriate methods of treatment. It is important that this system is socially acceptable and that it makes use of the correct channels to achieve technological levels that are accessible to people in and surrounding the community.

Primary prevention consists mainly of health promotion strategies to prevent illnesses from occurring. Health education is an example of this and forms a major component of primary health care.^33^ Participants in this study described the benefits of having rehabilitation services in primary health care centres, as they felt that through participation in vocational rehabilitation programmes and learning new skills, they would be able to either resume their previous worker role, or assume a new worker role. Donnelly et al.^34^ stated that there is a great need for the integration of rehabilitation services with primary health care centres. This is largely due to the need of the service being readily accessible to patients with more severe conditions, those who have unstable conditions and those requiring long term intervention. Therefore, in order for vocational rehabilitation programmes to be successfully implemented, it is required that primary health care facilities understand the importance of such programmes, so that they can motivate for its implementation. Horstman et al.^35^ argues that during vocational rehabilitation, the patient's main focus is typically to get healthy so that they may enter the open labour market, which becomes a distraction and takes the focus away from having TB**.**

#### Theme Four: engagement in social activities alleviates feelings of despondency

Fendt-Newlin and Webber^36^ describe a social network as having a close, supportive relationship with family and friends, as well as casual interactions with people within in the community. It was identified by a participant in the study that having the support from family and friends is vital for a successful recovery. Fendt-Newlin and Webber^36^ emphasise the importance of having a good social environment and close relationships (either intimate or platonic where a person feels close to another) as a key role in enhancing physical and mental health. This is because having good social relationships is not only important for one's psychological and emotional well-being, but also directly impacts one's physical well-being.^37^ From the statements of the participants and through the description of their experiences, this study revealed that patients feel less of a burden towards others when they are provided with emotional support, as well as when they realise that they are not alone in their rehabilitation process. It was identified in the current study that through developing good, trusting relationships, that patients became more comfortable and willing to speak about their thoughts and feelings.

## Limitations to the study

The recruitment of PTB survivors who returned to work was challenging. Only one male participant was recruited in the PTB and MDR-TB survivors’ subgroup. Despite concerted efforts to recruit from both genders, it was difficult to identify male participants. The breadwinner role is culturally often assigned to males and thus the limited attention paid to gender intersectionality was a limitation in the present study.

## Conclusion

Through the findings of this study, barriers and facilitators of return to work were identified in the rehabilitation of patients with PTB and MDR-TB survivors’ experiences. The barriers identified in the present study included “A sense of disbelief in one's own potential” This sense of disbelief stemmed from the poor motivation and belief the participants had in themselves after having suffered from PTB and MDR-TB. When having to return to their homes after hospitalisation, it was identified that the contextual environment in which the PTB and MDR-TB survivors found themselves in, negatively influenced their return to their previous worker role, or the pursuit of a new worker role. This was mainly due to the poor socio-economic factors which surrounded them. The facilitators that were highlighted in this study include; “The future of OT services in TB rehabilitation”, “Engagement in activities alleviates feelings of despondency”, and “Promoting a holistic model”. The importance of OT services in TB rehabilitation emerged through the perception of the rehabilitation care specialists as they identified the lack of public rehabilitation services that focus on work rehabilitation. They discussed how beneficial it would be for the patient suffering from PTB or MDR-TB to access work related intervention programmes in order to re-engage with their previous worker role or provide an opportunity to gain a new skill. Another facilitator that was highlighted in this study focused on the fact that through the engagement of meaningful activities, PTB and MDR-TB patients showed positive signs towards their improved overall well-being.
